# Association of preoperative flexion contracture and limited knee flexion with short-term patient-reported outcomes after medial unicompartmental knee arthroplasty

**DOI:** 10.2340/17453674.2026.46721

**Published:** 2026-09-09

**Authors:** Tomofumi KINOSHITA, Kristine Ifigenia BUNYOZ, Kristian R L MORTENSEN, Christian Bredgaard JENSEN, Christian S NIELSEN, Kirill GROMOV, Anders TROELSEN

**Affiliations:** 1 Department of Orthopaedic Surgery, Copenhagen University Hospital Hvidovre, Copenhagen, Denmark; 2 Department of Clinical Medicine, Copenhagen University, Copenhagen, Denmark; 3 Department of Orthopaedic Surgery, Ehime University Graduate School of Medicine, Ehime, Japan

## Abstract

**Background and purpose:**

We aimed to clarify whether preoperative flexion contracture (FC) and limited knee flexion in patients with anteromedial osteoarthritis (OA) were associated with postoperative outcomes in mobile-bearing medial unicompartmental knee arthroplasty (UKA).

**Methods:**

We retrospectively evaluated all patients who underwent mobile-bearing medial UKA. Preoperative extension and flexion angles, and the Oxford Knee Score (OKS) and Forgotten Joint Score-12 (FJS) recorded preoperatively and at 3, 12, and 24 months, were assessed. Patients were categorized by preoperative extension (no FC, < 10°, ≥ 10°) and flexion angles (< 120°, ≥ 120°). Changes in OKS and FJS were compared using general linear models with adjustment for age, sex, body mass index, and preoperative scores.

**Results:**

1,199 patients were included. Preoperatively, 85% of patients had full extension, 12% had < 10° FC, and 3.1% had ≥ 10° FC. Regarding flexion, 40% had < 120°, while 60% had ≥ 120°. At 12 months, the < 10° FC group showed greater improvements than the ≥ 10° FC group in OKS (adjusted mean difference, 5.5 points; 95% confidence interval [CI] 0.9–10.2; P = 0.01) and FJS (17.0 points; CI 1.0–33.0; P = 0.03). The < 120° flexion group showed greater FJS improvement than the ≥ 120° flexion group (adjusted mean difference, 4.8 points; CI 0.1–9.6; P = 0.045). At 24 months, the CIs for all between-group differences included no difference.

**Conclusion:**

Mild preoperative FC and mild-to-moderately limited knee flexion were not associated with inferior outcomes. Patients with more pronounced FC showed slower recovery but still achieved the same increase in patient-reported outcome measures (PROMs) as those with mild FC.

The indication for unicompartmental knee arthroplasty (UKA) has traditionally been considered narrow in patients with limited range of motion (ROM) [1]; however, recent studies have reported favorable outcomes in selected patients with flexion contracture (FC), although in general that around 23% have pain 1 year after surgery [2–4]. The acceptable degree of preoperative ROM limitation, including both FC and limited knee flexion, remains unclear and continues to challenge patient selection. Preoperative FC often persists after surgery [3,5], and severe residual contracture may negatively affect clinical outcomes [6,7] underscoring the need for more robust evidence.

Few studies have directly examined whether preoperative ROM limitations affect postoperative outcomes after UKA, and the degree of flexion limitation compatible with favorable UKA outcomes remains unclear.

It is clinically important to determine the extent of ROM restriction, particularly FC and limited knee flexion, that can be accepted in anteromedial OA. Clarifying these thresholds may help improve patient selection for medial UKA and contribute to better postoperative recovery and patient-reported outcomes.

We aimed to investigate the influence of preoperative FC and limited knee flexion on postoperative clinical outcomes in patients with anteromedial OA undergoing Oxford medial UKA.

## Methods

This study was reported in accordance with the Strengthening the Reporting of Observational Studies in Epidemiology (STROBE) guidelines [8]. Patients with anteromedial OA who underwent primary cementless UKA between August 2016 and November 2024 and who provided informed consent were retrospectively identified and included. The exclusion criteria were absence of preoperative ROM data, rheumatoid arthritis, history of ipsilateral knee surgery (including previous intra-articular or proximal tibial fracture surgery and meniscectomy resulting in secondary osteoarthritis), and reoperation.

### Surgical method

All surgeries were performed using the Oxford Phase 3 mobile-bearing unicompartmental knee arthroplasty system (Zimmer Biomet, Oxford, UK) with Microplasty instrumentation through a minimally invasive medial parapatellar approach, without eversion of the patella. Surgery was done in accordance with the manufacturer’s manual; the tibial resection was intended to have a 7° posterior slope in the sagittal plane. Before assessing intraoperative balance with trial components, osteophytes were removed to prevent impingement. Posterior, medial, and notch osteophytes of the femur and anterior tibial osteophytes were excised. No additional soft-tissue releases, including the medial collateral ligament or posterior capsule, were performed.

### Knee extension and flexion angle

Based on the preoperative maximum extension and flexion angles of the knee, patients were classified into subgroups as follows: (i) according to the preoperative maximum extension angle, into a no-FC group, < 10°-FC group (1–9 °) and a ≥ 10°-FC group, and (ii) according to the preoperative maximum flexion angle, into < 120°-flexion-angle group and ≥ 120°-flexion-angle group. As no patient had severe preoperative flexion limitation (< 90°), a threshold of 120° was used to distinguish between relatively limited and preserved preoperative flexion. Preoperative knee extension and flexion angle were obtained from routine clinical assessments performed by the treating orthopedic surgeons and recorded in 5° increments based on visual estimation.

### Patient-reported outcome measurement (PROMs)

The Oxford Knee Score (OKS) and Forgotten Joint Score-12 (FJS) were used to assess clinical outcomes preoperatively and at 3 months, 12 months, and 24 months following UKA [9,10]. The OKS ranges from 0 to 48 and the FJS from 0 to 100, with higher scores indicating better outcomes for both measures. The MCID was considered to be 5 points for the OKS and 12.5 points for the FJS [11,12].

Furthermore, the changes in OKS and FJS from the preoperative baseline were calculated at each postoperative time point. Comparisons of these outcome measures between the respective groups were conducted.

### Statistics

Patients with missing PROM data were not excluded from the study cohort; instead, the available-case analyses were performed using available cases at each time point (pairwise exclusion of missing values). Changes in OKS and FJS were compared using a general linear model with Bonferroni-adjusted post hoc tests for the FC groups and pairwise comparisons of estimated marginal means for the flexion angle groups. Adjusted mean differences with 95% confidence intervals (CI) were calculated after controlling for age, sex, body mass index (BMI), and preoperative baseline scores.

The primary analyses evaluated changes in OKS and FJS according to the 2 exposure classifications: preoperative FC and preoperative flexion angle. Pairwise comparisons between groups at individual postoperative time points were considered secondary comparisons and were adjusted using the Bonferroni method. Given the evaluation of multiple exposure classifications, PROM outcomes, and postoperative time points, isolated findings at individual time points were interpreted cautiously because of the potential increased risk of type I error due to multiplicity.

The extent of missing PROM data at each time point and its distribution across the comparison groups were examined descriptively. The primary available-case analyses may be biased if the probability of missingness is related to unobserved outcomes. Therefore, as a sensitivity analysis addressing missing outcome data, linear mixed-effects models were fitted using all available repeated measurements of OKS and FJS. The models included group, time, the group-by-time interaction, sex, age, and BMI as fixed effects, with an unstructured covariance structure for repeated measurements. The mixed-effects models used all available outcome observations and assumed that data was missing at random. All analyses were 2-tailed, and P < 0.05 was considered statistically significant. Statistical analyses were conducted using SPSS Statistics version 28.0 (IBM Corp, Armonk, NY, USA).

### Ethics, data sharing plan, funding, use of AI, and disclosures

This single-center cohort study was conducted in accordance with the Declaration of Helsinki and was approved by the Knowledge Centre on Data Protection Compliance, Capital Region of Denmark. The data supporting the findings of this study is available from the corresponding author upon reasonable request. No funding was received for this study. ChatGPT (OpenAI) was used for language editing and improving the clarity of the manuscript. The authors reviewed and approved all changes and take full responsibility for the content of the manuscript. Complete disclosure of interest forms according to ICMJE are available on the article page, doi: 10.2340/17453674.2026.46721

## Results

### Distribution of preoperative knee extension and flexion angle

**Figure 1:**
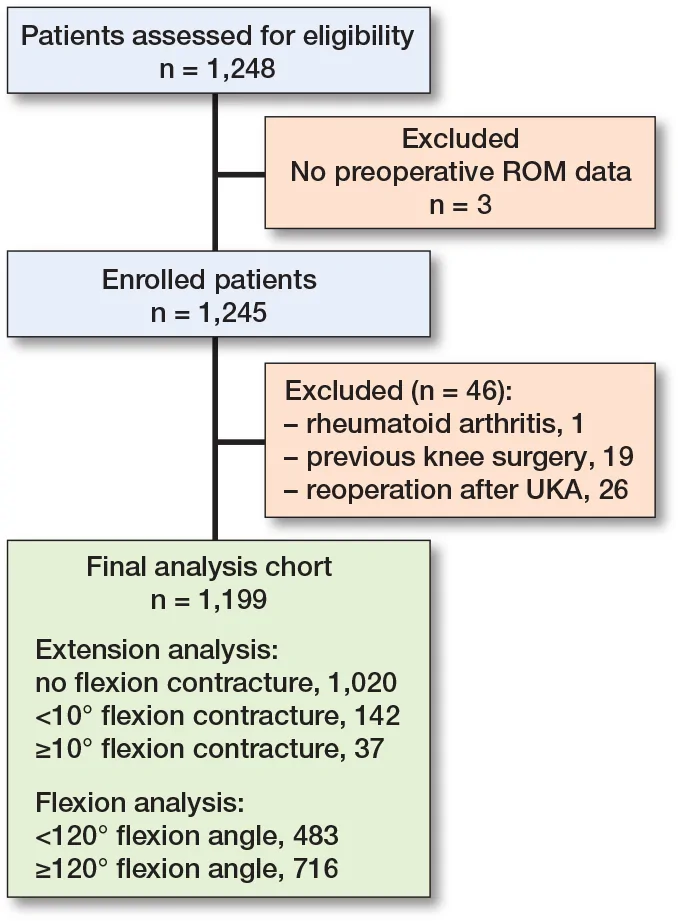
Flow diagram of patient inclusion and exclusion. ROM: range of motion; UKA: unicompartmental knee arthroplasty.

**Figure 2:**
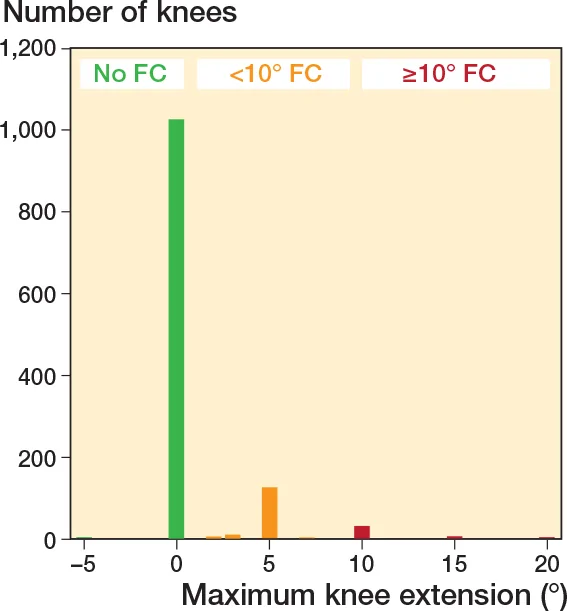
Distribution of preoperative maximum knee extension angle in patients undergoing medial unicompartmental knee arthroplasty

**Figure 3:**
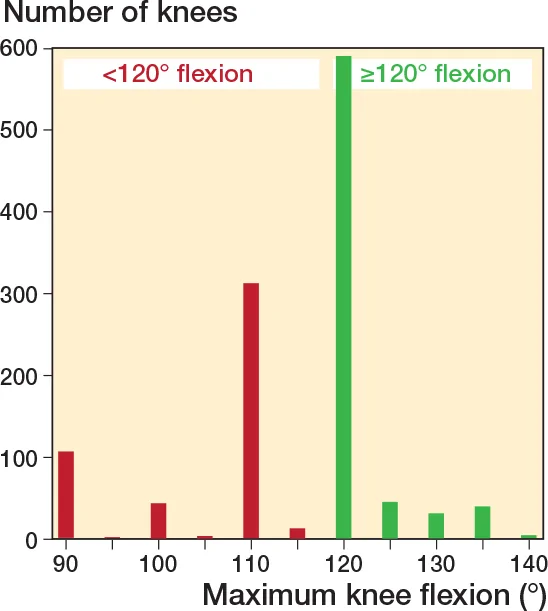
Distribution of preoperative maximum flexion angle in patients undergoing medial unicompartmental knee arthroplasty.

The exclusion criteria were absence of preoperative ROM data (n = 3), rheumatoid arthritis (n = 1), history of ipsilateral knee surgery (including previous intra-articular or proximal tibial fracture surgery [n = 3] and meniscectomy resulting in secondary osteoarthritis [n = 16]), and reoperation (n = 26). In total, 1,199 patients were included (Figure 1). Preoperatively, 1,020 (85%) of patients had full extension, while 142 (12%) patients were classified into the < 10°-FC group and 37 (3.1%) into the ≥ 10°-FC group (Figure 2). 2 knees demonstrated 5° of hyperextension. 6 knees showed flexion contracture of ≥ 15° (5 with 15° and 1 with 20°). Regarding knee flexion angle, 483 (40%) patients were classified into the < 120°-flexion group and 716 (60%) patients into the ≥ 120°-flexion group. No cases demonstrated a maximum flexion angle of < 90° (Figure 3). Preoperative patient characteristics are presented in Tables 1 and 2. Preoperative OKS and FJS were similar across the groups.

**Table 1:** Preoperative patient characteristics according to preoperative knee extension angle. Values are mean (SD) or as specified

Preoperative data	No FC group	< 10° FC group	≥ 10° FC group
Female sex, n (%)	590 (58)	67 (47)	17 (45)
Male sex, n (%)	430 (42)	75 (53)	20 (54)
Age, years	67 (9.4)	69 (9.0)	69 (8.6)
Height, cm	171 (10)	173 (10)	172 (10)
Weight, kg	89 (18)	94 (20)	88 (17)
Maximum knee extension, °	0.0 (0.2)	4.8 (0.8)	11 (2.3)
Maximum knee flexion, °	115 (11)	113 (9.1)	117 (11)
Oxford Knee Score	23.5 (7.3)	23 (7.5)	23 (7.7)
Forgotten Joint Score-12	18 (16)	19 (14)	21 (20)

FC: flexion contracture; SD: standard deviation.

**Table 2:** Preoperative patient characteristics according to preoperative knee flexion angle. Values are mean (SD) or as specified

Preoperative data	< 120°- flexion group	≥ 120°- flexion group
Female sex, n (%)	302 (63)	372 (52)
Male sex, n (%)	181 (37)	344 (48)
Age, years	67 (9.4)	68 (9.2)
Height, cm	170 (10)	172 (10)
Weight, kg	92 (18)	88 (18)
Maximum knee extension, °	1.3 (2.8)	0.6 (2.1)
Maximum knee flexion, °	105 (8.4)	122 (4.4)
Oxford Knee Score	23 (7.4)	24 (7.3)
Forgotten Joint Score-12	18 (15)	18 (16)

SD: standard deviation.

### Mean changes in OKS and FJS according to preoperative knee extension angle

**Figure 4:**
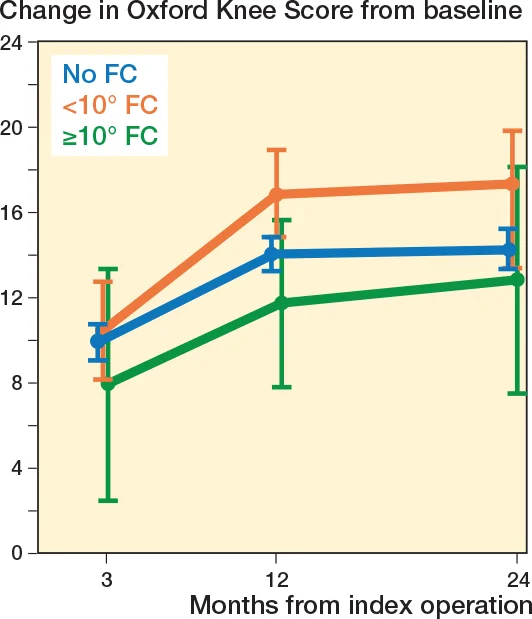
Comparison of changes in Oxford Knee Score from preoperative score among the no flexion contracture, < 10° flexion contracture, and ≥ 10° flexion contracture groups.

At 12 months postoperatively, the < 10°-FC group showed greater improvement in OKS than the ≥ 10°-FC group, with an adjusted mean difference of 5.5 points (CI 0.9–10.2; P = 0.01; Figure 4 and Table 3). At 24 months, the corresponding adjusted mean difference was 5.0 points (CI −0.7 to 10.6; P = 0.1).

**Table 3:** Pairwise comparisons of changes in Oxford Knee Score and Forgotten Joint Score-12 among the no flexion contracture group, < 10°-FC group, and ≥˘10°-FC group

Outcome Time	Adjusted ^a^ mean difference (CI)	P value ^b^	Mean difference (CI)	P value ^b^
Oxford Knee Score				
3 months				
No FC vs < 10°FC	0.0 (−2.6 to 2.6)	> 0.9	−0.5 (−3.4 to 2.4)	> 0.9
No FC vs ≥ 10°FC	0.8 (−4.4 to 6.0)	> 0.9	2.0 (−3.8 to 7.8)	> 0.9
< 10°FC vs ≥ 10°FC	0.8 (−4.9 to 6.4)	> 0.9	2.5 (−3.8 to 8.8)	> 0.9
12 months				
No FC vs < 10°F	−2.4 (−4.8 to 0.0)	0.05	−2.8 (−5.5 to −0.1)	0.04
No FC vs ≥ 10°FC	3.2 (−1.1 to 7.4)	0.2	2.4 (−2.5 to 7.2)	0.7
< 10°FC vs ≥ 10°FC	5.5 (0.9 to 10.2)	0.01	5.2 (−0.2 to 10.6)	0.06
24 months				
No FC vs < 10°FC	−2.6 (−5.4 to 0.2)	0.08	−3.0 (−6.1 to 0.0)	0.05
No FC vs ≥ 10°FC	2.4 (−2.8 to 7.5)	0.8	1.4 (−4.3 to 7.2)	> 0.9
< 10°FC vs ≥ 10°FC	5.0 (−0.7 to 10.6)	0.1	4.5 (−1.8 to 10.7)	0.3
Forgotten Joint Score-12				
3 months				
No FC vs < 10°FC	−3.9 (−12.2 to 4.4)	0.8	−3.9 (−12.6 to 4.7)	0.8
No FC vs ≥ 10°FC	7.7 (−8.3 to 23.8)	0.7	9.4 (−7.5 to 26.2)	0.5
< 10°FC vs ≥ 10°FC	11.6 (−5.8 to 29.1)	0.3	13.3 (−5.2 to 31.7)	0.3
12 months				
No FC vs < 10°FC	−7.7 (−16.0 to 0.6)	0.08	−7.2 (−16.1 to 1.7)	0.2
No FC vs ≥ 10°FC	9.4 (−5.1 to 23.8)	0.3	9.1 (−6.5 to 24.7)	0.5
< 10°FC vs ≥ 10°FC	17.0 (1.0 to 33.0)	0.03	16.3 (−1.1 to 33.6)	0.07
24 months				
No FC vs < 10°FC	−5.6 (−15.0 to 3.7)	0.4	−5.2 (−15.0 to 4.7)	0.6
No FC vs ≥ 10°FC	2.6 (−14.4 to 19.6)	> 0.9	1.7 (−16.4 to 19.8)	> 0.9
< 10°FC vs ≥ 10°FC	8.2 (−10.5 to 26.9)	0.9	6.8 (−13.1 to 26.7)	> 0.9

CI: 95% confidence interval; FC: flexion contracture.

a Adjusted values were obtained by controlling for age, sex, body mass index, and preoperative baseline score.

b Bonferroni adjusted.

**Figure 5:**
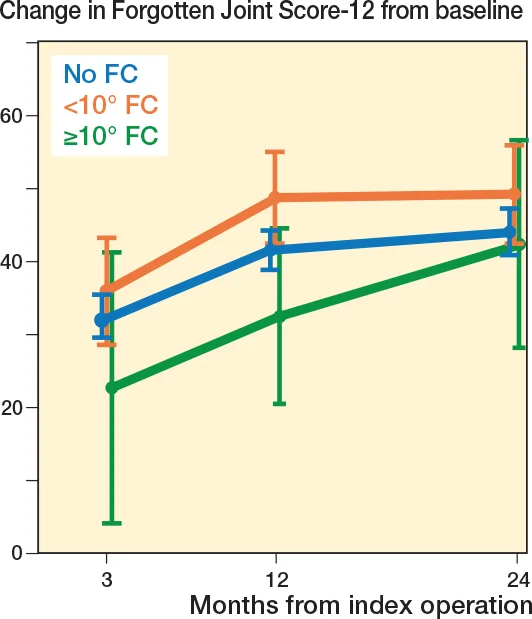
Comparison of changes in Forgotten Joint Score-12 from preoperative score among the no flexion contracture, < 10° flexion contracture, and ≥ 10° flexion contracture groups.

At 12 months postoperatively, the < 10°-FC group showed greater improvement in FJS than the ≥ 10°-FC group, with an adjusted mean difference of 17.0 points (CI, 1.0– 33.0; P = 0.03; Figure 5 and Table 3). At 24 months, the corresponding adjusted mean difference was 8.2 points (CI −10.5 to 26.9; P = 0.9).

### Mean changes in OKS and FJS according to preoperative knee flexion angle

**Figure 6:**
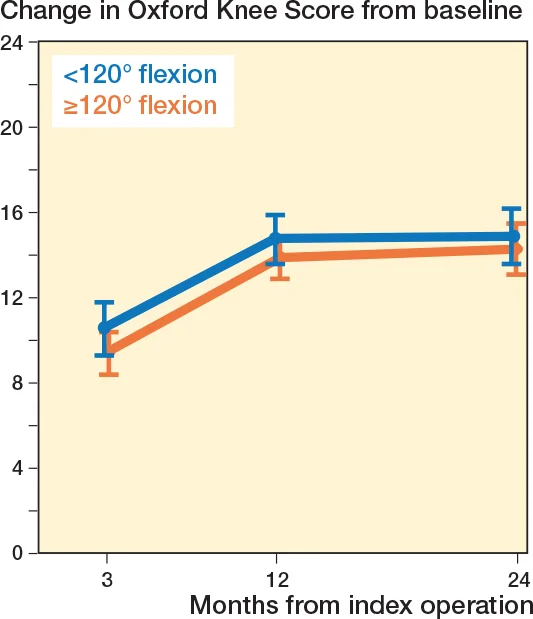
Comparison of changes in Oxford Knee Score from preoperative score among < 120° flexion angle group and ≥ 120° flexion angle group.

At 12 months, the adjusted mean difference in OKS improvement between the < 120° and ≥ 120° flexion-angle groups was 1.0 point (CI −0.4 to 2.4; P = 0.2). At 24 months, the adjusted mean difference was 0.6 points (CI −1.1 to 2.2; P = 0.5; Figure 6 and Table 4).

**Table 4:** Pairwise comparisons of changes in Oxford Knee Score and Forgotten Joint Score-12 between the preoperative < 120° flexion group and ≥ 120° flexion group

Outcome Time	Adjusted ^a^ mean difference (CI)	P value ^b^	Mean difference (CI)	P value ^b^
Oxford Knee Score				
3 months				
< 120° vs ≥ 120°	0.7 (−0.8 to 2.2)	0.4	1.1 (−0.5 to 2.7)	0.2
12 months				
< 120° vs ≥ 120°	1.0 (−0.4 to 2.4)	0.2	0.9 (−0.7 to 2.4)	0.3
24 months				
< 120° vs ≥ 120°	0.6 (−1.1 to 2.2)	0.5	0.6 (−1.3 to 2.4)	0.5
Forgotten Joint Score-12				
3 months				
< 120° vs ≥ 120°	3.7 (−1.0 to 8.4)	0.1	2.9 (−1.9 to 7.8)	0.2
12 months				
< 120° vs ≥ 120°	4.8 (0.1 to 9.6)	0.045	2.0 (−3.0 to 6.9)	0.4
24 months				
< 120° vs ≥ 120°	1.4 (−4.2 to 7.0)	0.6	−1.7 (−7.3 to 4.0)	0.6

For abbreviations, see Table 3.

**Figure 7:**
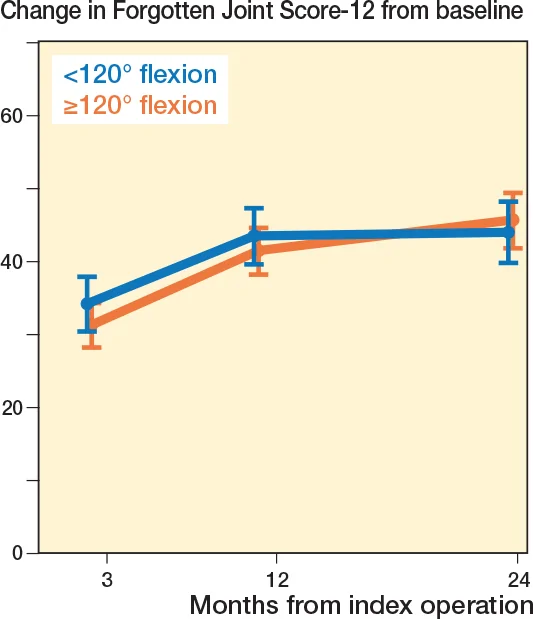
Comparison of changes in Forgotten Joint Score-12 from preoperative score among < 120° flexion angle group and ≥ 120° flexion angle group.

At 12 months, the < 120°-flexion group showed greater improvement in FJS than the ≥ 120°-flexion group, with an adjusted mean difference of 4.8 points (CI 0.1– 9.6; P = 0.045). At 24 months, the adjusted mean difference was 1.4 points (CI −4.1 to 7.0; P = 0.6; Figure 7 and Table 4).

### Sensitivity analyses using linear mixed-effects models

In sensitivity analyses using linear mixed-effects models including all available repeated measurements, both OKS and FJS improved over time in the FC and flexion angle analyses (all P < 0.001). No group-by-time interactions were observed for OKS or FJS in either the FC analysis (P = 0.6 and P = 0.9, respectively; Table 5) or the flexion angle analysis (P = 0.6 and P = 0.7, respectively; Table 6), indicating no clear evidence of differential longitudinal changes between the respective groups.

**Table 5:** Adjusted estimated marginal means of Oxford Knee Score and Forgotten Joint Score-12 from linear mixed-effects models according to preoperative flexion contracture group in medial UKA

Outcome Time	Adjusted mean (CI)		
	No FC	< 10° FC	≥ 10° FC
Oxford Knee Score			
Preoperative	24 (23–24)	23 (22–25)	24 (21–27)
3 months	34 (33–35)	34 (32–35)	33 (30–36)
12 months	38 (38–39)	39 (37–40)	36 (33–39)
24 months	38 (37–39)	39 (37–41)	36 (33–39)
Forgotten Joint Score-12			
Preoperative	19 (17–20)	20 (16–23)	22 (16–29)
3 months	52 (50–54)	54 (49–58)	51 (42–60)
12 months	61 (59–62)	62 (58–67)	59 (50–68)
24 months	62 (60–64)	65 (60–71)	62 (52–72)

For abbreviations, see Table 3.

**Table 6:** Adjusted estimated marginal means of OKS and FJS from linear mixed-effects models according to preoperative knee flexion angle group in medial UKA

Outcome Time	Adjusted mean (CI)	
	Flexion < 120°	Flexion ≥ 120
Oxford Knee Scor		
Preoperative	24 (23–24)	24 (23–25)
3 months	34 (33–35)	34 (33–34)
12 months	38 (38–39)	38 (37–39)
24 months	38 (37–39)	38 (37–39)
Forgotten Joint Score-12		
Preoperative	20 (18–22)	18 (17–20)
3 months	54 (51–57)	51 (49–53)
12 months	63 (60–65)	60 (57–62)
24 months	63 (61–66)	62 (59–64)

For abbreviations, see Table 3.

## Discussion

We aimed to clarify whether preoperative FC and limited knee flexion in patients with anteromedial osteoarthritis are related to postoperative outcomes in mobile-bearing medial UKA.

We found that preoperative FC and limited knee flexion were not associated with inferior outcomes following medial UKA. Regarding FC, the ≥ 10°-FC group showed smaller improvements in OKS and FJS at 12 months; however, these differences were no longer observed at 24 months. Although the mean improvement in the ≥ 10°-FC group remained slightly lower than that of the other groups, patients still demonstrated substantial improvement compared with their preoperative status. The adjusted between-group difference in OKS at 12 months (5 points) was comparable to the reported MCID for the OKS [11], suggesting that the delayed recovery observed in patients with ≥ 10° FC may have been clinically relevant during the early postoperative period. Similarly, for limited knee flexion, a difference in FJS improvement was observed between the 2 groups at 12 months, but this difference had disappeared by 24 months. Improvements in OKS showed no significant differences at any time point.

Previous studies have suggested that residual severe FC may negatively affect outcomes after UKA [6,7]. Goh et al. suggested that severe preoperative FC may not necessarily compromise acceptable outcomes after UKA, although postoperative motion was slightly limited [3]. Similarly, Wignadasan et al. reported that in patients with preoperative FC ≥ 10° (median, 10°), FC improved to a median of 5° at 12 months after UKA [13]. These findings indicate that mild FC can improve postoperatively, although full extension may not always be achieved. In the present study, patients with preoperative FC ≥ 10° also achieved favorable outcomes, with mean improvements of 12.8 in OKS and 42.4 in FJS at 24 months postoperatively, further supporting the suitability of UKA in cases with mild FC. However, in the present series, only 1 patient exhibited an FC of ≥ 20°, and truly severe cases were not sufficiently represented. Therefore, further studies are needed to determine the degree of severe FC that can be safely tolerated in medial UKA for patients with anteromedial OA.

No studies have clearly defined the acceptable degree of preoperative flexion limitation in UKA. Previous research has shown that postoperative flexion after UKA is influenced by preoperative flexion [14] and that postoperative flexion, in turn, affects clinical outcomes [15]. Hiranaka et al. reported that knees with smaller preoperative flexion angles tended to gain motion after UKA, whereas those with greater flexion tended to lose it. They identified a threshold of approximately 135°, suggesting that UKA can improve flexion in patients starting below this level [16]. Nagata et al. compared patients undergoing simultaneous bilateral arthroplasty with UKA on 1 side and TKA on the other with patients undergoing bilateral TKA. Postoperative flexion angle was significantly greater after UKA, even after adjusting for preoperative flexion angle [17]. This finding highlights the advantage of UKA in achieving greater postoperative flexion; however, because postoperative flexion is still influenced by preoperative flexion, the acceptable degree of preoperative flexion limitation remains uncertain. In the present study, patients with preoperative flexion angles of around 90° to 120° also achieved satisfactory short-term outcomes. Taken together with previous findings, these results suggest that moderate flexion limitation does not necessarily preclude favorable outcomes after medial UKA.

FC and limited knee flexion are commonly present together in patients with knee osteoarthritis, although their underlying mechanisms differ. FC primarily develops as pain and inflammation restrict extension, and posterior femoral osteophytes in the medial compartment, as well as anterior tibial osteophytes, can further mechanically block full extension [18,19]. In contrast, limited knee flexion is mainly attributed to mechanical impingement caused by osteophytes [20] or synovial hypertrophy [21], as well as pain-related inhibition of deep flexion [22]. Importantly, in appropriately selected patients with anteromedial OA, removing impinging osteophytes can improve the arc of motion by alleviating mechanical blockage. These factors may be addressed intraoperatively through posterior osteophyte removal, notch or medial osteophyte resection combined with postoperative pain control and improved synovial condition. In our study, patients with mild FC or limited knee flexion achieved favorable clinical outcomes. These findings suggest that such ROM limitations do not preclude satisfactory results when adequate bone resection and balancing are achieved during medial UKA in patients with anteromedial OA.

### Limitations

No patients with severe FC were included; the greatest degree of preoperative FC in this cohort was 20°, seen in only 1 patient. Likewise, no patients had a preoperative flexion < 90°. However, such cases are very rare within the appropriate indications for Oxford UKA, namely anteromedial OA, and this limitation is therefore unlikely to diminish the clinical relevance of our findings. ROM was assessed only preoperatively, and postoperative ROM data was not evaluated; thus, the results do not indicate whether patients achieved favorable postoperative motion. Further prospective studies should investigate whether persistent postoperative FC and limited knee flexion are associated with clinical outcomes. In addition, ROM measurements were obtained as part of routine clinical practice by multiple clinicians, and interobserver reliability was not evaluated. Nevertheless, the findings suggest that, even in the presence of FC or limited knee flexion, meaningful improvements in pain and function can still be expected. In addition, PROMs were not available for all patients at all follow-up points, and the analyses were conducted based on the available data at each time point, introducing potential selection and information bias. Further, the follow-up period was limited to the short term, precluding conclusions regarding long-term outcomes. Moreover, the number of patients with ≥ 10° FC was relatively small, which may have limited the statistical power to detect subtle differences between groups.

### Conclusion

Mild preoperative FC and mild-to-moderate limited knee flexion were not associated with inferior outcomes. Patients with more pronounced FC showed slower recovery but still achieved substantial benefit from surgery.

*In perspective*, our findings demonstrate that UKA can achieve satisfactory outcomes even in patients with mild FC, and further provide evidence that favorable results can be obtained even in those with limited preoperative knee flexion. In appropriately selected patients with anteromedial OA , mild FC and mild-to-moderate limited knee flexion should not be considered contraindications to medial UKA.

## Notes

TK: Methodology, formal analysis, investigation, writing – original draft, writing – review & editing; CBJ, CSN, KIB, KRLM: Investigation, formal analysis, writing – review & editing; KG: methodology, writing – review & editing; AT: Conceptualization, methodology, writing – review & editing.

Handling co-editors: Marianne Westberg and Robin Christensen

*Acta* thanks Rutger van Geenen and other anonymous reviewers for help with peer review of this manuscript.
